# Supplementary material for: Are carbon emissions trading and green financial instruments synergistic? -Comprehensive quantitative research based on content analysis
Source: PLoS One. 2024 Mar 7;19(3):e0298601. doi: 10.1371/journal.pone.0298601 (PMC10919601; doi:10.1371/journal.pone.0298601)
Supplement: S1 Data — (ZIP) [file pone.0298601.s001.zip › Data/地级市Z_F工作报告.zip!/║╙─╧/║╫▒┌╩╨/└┤╫╘╣1⁄2╓┌║┼í╛┬φ┐╦╩2╛▌═°í┐_2016.docx]

各位代表：

　　现在，我代表市人民政府，向大会报告工作，请予审议，并请各位政协委员和其他列席人士提出意见。

　　一、“十二五”取得成就及“十三五”主要目标

　　过去的五年，我们走过的历程很不平凡。面对严峻复杂形势和一系列风险挑战，全市人民深入贯彻落实省委、省政府和市委决策部署，主动适应新常态，围绕在全省率先全面建成小康社会，克难攻坚，务实重干，较好完成了“十二五”规划主要目标任务，经济社会发展取得了令人鼓舞的成就。

　　经济实力大幅提升。全市地区生产总值达到713亿元，是2010年的1.6倍。预计人均生产总值接近4.5万元，是2010年的1.5倍。完成规模以上工业增加值444.2亿元，是2010年的1.7倍。一般公共预算收入、支出分别达到52.9亿元和114.4亿元，是2010年的2.4倍和1.9倍。金融机构存款余额536.8亿元，是2010年的1.9倍。

　　产业转型步伐加快。“互联网+”、电子商务、休闲旅游、新型物流等新业态快速发展，第三产业完成增加值占生产总值的比重由18%提高到25.2%。高新技术产业增加值占规模以上工业增加值的比重由3.8%提高到29.9%，煤炭行业占比由19.8%下降到8.3%，能源资源型产业偏重状况得到持续改善。粮食总产123.6万吨，比2010年增加12万吨，被确定为全国整建制推进粮食高产创建试点市、整市建制国家现代农业示范区等。

　　城乡发展趋于协调。预计城镇化率达到55.66%、继续保持全省前列。单元组合型城市中心带动、协调联动能力持续提升，中心城区、老城区、两县县城面貌和功能持续改善，市区建成区面积增加到68.7平方公里。农村环境综合整治成效明显，成功创建一批省级以上重点镇、美丽乡村、传统村落、生态文化村、水美乡村。实现了所有县区与中心城区、所有建制村之间都有一条等级公路连通，200辆新能源公交车和千余辆公共自行车投入使用，市民出行更加方便。被确定为全国质量强市示范市、全国海绵城市建设试点市、全国首家国土空间优化发展实验区等。

　　载体支撑更加有力。产业集聚区和园区实现了上星晋位，规模以上工业增加值占全市的67.9%，成为工业增长的主阵地。城乡一体化示范区三产融合发展格局基本形成，商务中心区和特色商业区不断发展壮大、建成区面积达到5.1平方公里。鹤壁海关、出入境检验检疫局和一批特色出口加工基地建成投用，综合保税区上报国家待批。预计去年全社会固定资产投资完成700亿元、是2010年的2.5倍，累计新建改建农村公路806公里，日供水能力提高到40.2万吨，天然气用户接近50万人，供热覆盖面积增加到1200万平方米。

　　发展后劲不断增强。开放招商、项目建设目标完成进度和增速保持全省先进水平，累计引进市外资金、新开工项目、竣工项目和完成投资额分别是“十一五”的2倍、2.3倍、1.3倍和3.1倍，进出口总值达到3.4亿美元、是2010年的2.3倍，南水北调、石武客运专线、晋豫鲁铁路鹤壁段和鹤淇电厂等316个重点项目竣工。企业服务扎实有效，累计完成各类技能培训29.1万人次、新增建设用地6万余亩，金融机构贷款余额是2010年的1.8倍，设立了百亿元产业发展投资基金和3.2亿元中小企业还贷周转金，“助保贷”规模扩大到3.6亿元，与一批域外金融机构、省重要投融资集团建立了战略关系、开展了合作业务，23家企业在新三板或区域性股权交易市场挂牌。

　　动力活力持续释放。政府职能转变和机构改革基本完成，行政审批事项和时限大幅压缩，被确定为省行政审批标准化试点市，出台了支持县区、产业集聚区等系列有针对性的政策措施，调整优化了管理体制机制，探索组建了4个重点产业集团。创业创新热情高涨，市场主体增长86%、注册资本增长438.5%。科技创新全面深化，与全国49家科研院校、12家省级以上学会协会建立了合作关系，建成省级以上创新研发平台67家，荣获国家科技进步一等奖1项、省部级科技进步奖27项。成功创建国家可持续发展试验区、全国创新驱动助力工程示范市等。

　　生态环境得到优化。城市环境空气优良天数、淇河水质保持全省第一，森林覆盖率达到32.2%、较2010年提高7.4个百分点，成功创建国家森林城市等。节能减排提前一年完成“十二五”主要污染物总量减排任务，是首个通过验收的国家循环经济标准化试点市、首批国家循环经济示范市创建城市，被确定为国家节能减排财政政策综合示范市。

　　人民生活明显改善。财政用于民生投入五年累计363.1亿元、较“十一五”增加248亿元，占当期财政支出比例由61.8%提高到80.1%。城乡居民人均可支配收入分别是2010年的1.7倍和1.9倍。累计城镇新增就业20.6万人、农村劳动力转移就业新增12.3万人。企业退休人员养老金连年提高10%，城乡居民医保、人均基本公共卫生服务补助标准分别是2010年的3.2倍和2.7倍，城乡低保对象补助标准分别是2010年的2.1倍和2.9倍，农村五保对象集中、分散供养标准分别是2010年的1.9倍和2.3倍；累计开工保障性住房10.2万套、分配安置近4万套，改善了近10万人的住房条件。城乡免费义务教育全面实现，中小学生均公用经费标准分别是2010年的1.6倍和1.7倍，学前三年入园率、小学初中净入学率、高中毛入学率全省先进，新增高等院校2所，河南理工大学鹤壁工程技术学院实现本科招生，被评为省职教攻坚工作先进市。民俗文化节、文博会、樱花节、环淇河自行车赛等影响日益扩大，实现了常态化文艺演出和公益性文化服务场所免费开放，大运河浚县段、黎阳仓遗址入选世界文化遗产名录。人民医院综合病房楼等一批项目建成投用，对外合作办医实现重大进展，每千人口拥有床位数、医师数、护士数均高于全省、全国平均水平。盘石头水库三期移民搬迁基本完成，4.59万农村贫困人口稳定脱贫，改造农村危房2.7万多户，解决了31.6万农村居民、在校师生安全饮水问题和2.4万农户“低电压”问题。

　　五年来，我们切实加强民主法治建设，自觉接受人大法律和工作监督、政协民主监督、社会舆论监督，支持政协参政议政，1540件人大代表建议和政协提案全部办结；深入推进依法治市、依法行政，“六五”普法顺利完成，公众法治环境满意度居全省前列，被确定为全国法治城市创建活动先进单位。切实加强政府自身建设，深入落实中央八项规定和省、市实施意见，扎实开展党的群众路线教育实践活动、“三严三实”专题教育，持续深化反腐倡廉，专项治理懒政怠政为官不为行为，风清气正、干事创业的氛围更加浓厚。切实加强精神文明建设，开展系列公民道德建设和文明创建活动，“鹤壁好人”越来越多，涌现出一批全国先进典型。切实加强和创新社会治理，持续深化平安建设，着力抓好安全生产，依法改进信访工作，公众安全感和满意度保持全省前列。切实加强国防教育、国防后备力量及人民防空建设，推动军政军民团结深度融合发展，驻鹤解放军、武警部队和民兵预备役为地方经济社会发展做出重要贡献。民族宗教和外事侨务、对台、统计、档案、史志、地震、人防等工作取得新成绩。

　　各位代表，“十二五”时期是我市经济社会持续健康发展、综合实力大幅提升的五年，是转型升级深入推进、质量效益明显提高的五年，是城市形象愈发靓丽、知名度美誉度持续扩大的五年，是社会大局和谐稳定、人民生活越来越幸福的五年。这些成绩的取得，是省委、省政府和市委正确领导的结果，是市人大和市政协大力支持的结果，更是全市人民拼搏奉献的结果。在此，我代表市政府，向全市人民，向各民主党派、各人民团体和各界人士表示崇高的敬意，向关心支持鹤壁发展的各界朋友表示诚挚的感谢！

　　五年来，我们把提升求进作为工作基调，坚持一张蓝图绘到底，持续提升科学发展的理念、动力、举措、层次和质量，提高了城市竞争力和影响力；把统筹兼顾作为根本方法，全面推进经济、政治、文化、社会、生态文明建设，增强了工作的协调性和可持续性；把改革创新作为核心抓手，有效破解了影响发展的体制机制障碍和瓶颈制约，进一步激发了动力和活力；把打造优势作为关键举措，持续发挥比较优势、挖掘潜在优势、创造后发优势，实现了由局部优势向综合优势的加快转变；把奋勇争先作为价值导向，持续做精品创品牌成特色，国家级试点、示范达到35个；把凝聚合力作为源泉保障，形成了万众一心谋发展推发展的强大合力。这些经验是全市人民智慧的结晶，是一笔宝贵的精神财富，应当倍加珍惜并不断发扬光大。

　　各位代表，“十三五”时期是全面建成小康社会决胜阶段，我市仍处于可以大有作为的重要战略机遇期，处于爬坡过坎、升级突破的关键期，必须在已有基础上提升发展、决胜小康。根据市委八届十次全会精神，市政府编制了《鹤壁市国民经济和社会发展第十三个五年规划纲要（草案）》，已印发会议提请审议。

　　“十三五”时期经济社会发展的指导思想是：高举中国特色社会主义伟大旗帜，全面贯彻党的十八大和十八届三中、四中、五中全会精神，以马列主义、毛泽东思想、邓小平理论、“三个代表”重要思想、科学发展观为指导，深入贯彻习近平总书记系列重要讲话精神，认真落实中央“四个全面”战略布局和省委战略部署，坚持发展第一要务，牢固树立创新、协调、绿色、开放、共享发展理念，更加注重供给侧结构性改革，以提高发展质量和效益为中心，以提高发展质量和效益为中心，加快构建现代产业、现代农业、现代城乡、现代生态、现代治理新体系，统筹推进经济建设、政治建设、文化建设、社会建设、生态文明建设和党的建设，推动四化同步科学发展，在全省率先全面建成小康社会，加快建设更高水平的生态文明之城、活力特色之城、幸福和谐之城，开启现代化建设新征程。

　　主要预期目标是：在提高发展平衡性、包容性、可持续性基础上，经济发展速度质量优于全省，主要经济指标年均增速高于全省平均水平，在全省率先实现生产总值和城乡居民人均收入比2010年翻一番目标；新型城镇化水平明显提高，新农村建设取得积极成效，城乡一体发展走在全省前列；生态系统良性循环，文明程度全面提高，生态文明质量保持全省先进；居民收入与经济增长同步，公共服务和保障体系更加健全，人民幸福水平普遍提升；民主法治更加健全，基本制度体系更加成熟，社会大局和谐稳定。

　　各位代表，率先全面建成小康社会令人鼓舞、催人奋进。我们将以“十三五”规划纲要为行动指南，把握好以人为本、发展理念、深化改革、依法治市、党的领导原则，树立提升求进、创新发展、示范引领、厚植优势、勇争一流工作导向，如期完成“十三五”目标任务，努力再谱新篇、再创辉煌！

　　二、2015年工作回顾

　　去年是“十二五”收官之年。我们统筹推进改革发展稳定各项工作，较好完成了市十届人大二次会议确定的目标任务。初步核算，地区生产总值增长8%，规模以上工业增加值增长7.5%，固定资产投资增长17.7%，进出口总值增长5.1%，一般公共预算收入增长12.2%，社会消费品零售总额增长12.4%，居民消费价格指数上涨1.3%，居民人均可支配收入增长9.6%。

　　一年来，我们着力稳增长保态势。牢牢抓住提升工业、扩大投资、稳定房地产三个关键，实施了促进经济平稳健康发展、确保工业经济平稳较快增长、促进房地产市场持续健康发展等政策措施。围绕主导先导产业壮大、服务业发展、新业态新模式培育、载体平台建设、风险隐患化解等20项重点工作，实施了保增长稳运行若干重点专项工作方案等。改进提升了开放招商、项目建设、企业服务工作，150个重点项目完成投资占年度计划的103%，24个项目竣工、70个项目开工；引进省外资金、利用境外资金分别增长9%、15.4%；企业反映问题解决率96%，有效保障了企业正常生产经营。深入研判形势，及时化解苗头性倾向性问题，经济运行总体平稳、稳中向好。

　　我们着力调结构促转型。持续做强工业、做优农业、做大服务业，高新技术和电子信息产业增加值增速分别高出规模以上工业8.4和13.4个百分点，煤电化材一体化产业占比下降到39.3%；粮食总产实现十二连增”，农业现代化综合指数全省第一；第三产业增加值增长12%、高出工业4.7个百分点。浚县和宝山产业集聚区成功创建国家级、省级产业示范基地。积极发展“互联网+”新业态，与曙光、腾讯等知名互联网公司开展了合作，鹤壁城市云计算中心、中国鹤壁城市智慧平台投入使用，“互联网+民生”等多个新业态取得实实在在成效。

　　我们着力推改革促创新。确定重点改革事项163项，并选择25项重大改革事项以及10项重中之重改革事项精准发力，完成或基本完成127项。出台了加快科技创新创业驱动产业发展、深入推进科技协同创新等实施意见，制定大众创业万众创新政策措施30条，5个国字号创新研发平台挂牌运行，新增国家级博士后科研工作站2个，建成省级创业孵化园区1家，新登记市场主体同比增长19.5%，国立光电荣获全国创业专项大赛总决赛冠军。

　　我们着力夯基础创优势。加快绿色低碳循环发展，完成造林绿化24万亩，新增城市绿地面积333.8万平方米，城市环境空气质量优良天数237天，新开工67个节能减排和循环经济项目、20个竣工，预计全年万元生产总值能耗下降6%。科学推进城乡一体发展，编制了全国首家市一级的《优化国土空间开发格局规划纲要》，鹤淇一体化进程步伐加快，老城区成方连片改造基本完成，城乡一体化示范镇、美丽乡村示范带等建设顺利推进，50%以上村庄初步建立了长效保洁机制，首批100个村基本达到人居环境示范村标准。

　　我们着力惠民生防风险。一方面持续保障改善民生。全市民生支出占财政支出82.5%、高出全省5.1个百分点。重点民生工程顺利实施，一批关系群众切身利益的热点难点问题得到有效解决。城镇新增就业、城镇失业人员再就业、新增农村劳动力转移就业等任务超额完成，完成各类技能培训5.68万人次，动态消除了零就业家庭。社会保障实现扩面提标，居民健康档案建档率、新农合参合率居全省前列，在全省率先实施“失独”家庭救助和养老扶助金政策，为80岁以上高龄老人发放了津贴。开工建设保障性住房995套、基本建成8131套，改造农村危房5890户。34所城乡学校建设扎实推进，4个县区通过义务教育基本均衡发展验收。上海细胞治疗工程技术研究中心河南中心建成投用，精准医疗研究院挂牌成立。实施整村推进项目16个，7480人实现脱贫。省级文明城市创建全面启动，6家单位被评为全国文明单位、4个村镇被评为全国文明村镇。另一方面把平安建设和社会稳定作为第一责任。深化社会治安综合治理，严格落实安全生产责任制，依法依规解决群众利益诉求，防范化解非法集资等金融风险，社会大局安全稳定。

　　各位代表，我们也清醒认识到，经济社会发展还存在一些突出矛盾和问题：从长期看，主要是经济总量不大、服务业发展相对滞后、城乡发展不均衡，自主创新能力不强、公共服务水平不高、体制机制不活等；从当前看，受经济下行和要素供给趋紧影响，投资增长有所放缓，部分行业企业困难加剧，种粮比较效益下降，风险隐患和环境污染依然突出。同时，政府职能转变还不到位，个别公务人员存在作风不实、服务不优、效能不高、为政不廉等现象。对此，我们一定高度重视，认真解决。

　　三、2016年发展目标和重点工作

　　今年是“十三五”开局之年。做好今年的政府工作，我们将进一步坚定发展信心决心，优化发展思路举措，更加注重稳增长、调结构、惠民生、防风险综合平衡，更加注重培育发展新动能、改造提升传统动能，更加注重长短结合、综合施策、精准发力，持续提升发展质量和效益，为“十三五”开好局、起好步。

　　今年经济社会发展主要预期目标是：地区生产总值增长8.5%左右，力争实际发展中取得更好成绩，其中规模以上工业增加值增长9%左右；全社会固定资产投资增长16%左右；一般公共预算收入与生产总值增长同步；居民人均可支配收入高于经济增长速度；社会消费品零售总额增长12%左右；城镇新增就业3万人以上；居民消费价格涨幅3%左右；常住人口城镇化率提高1.3个百分点；完成国家节能减排财政政策综合示范市要求目标和省下达环境质量改善任务。

　　今年，着力做好以下工作：

　　（一）突出三项重点，增强发展后劲。坚持扩充增量与激活存量并重，落实完善重点产业发展推进机制，一条龙、一体化推进开放招商、项目建设和企业服务工作，以重点突破带动全局提升，巩固经济运行的好态势。一是提升开放招商实效。延续定向招商、精准招商、专业招商、以商招商等好经验好做法，更加注重引项目与引资金、引智力与引平台相结合，更加注重企业联合行业协会、中介组织进行整体招商，更加注重优质项目引进来和优势企业走出去，力争每个县区都在龙头企业引进、产业集群化链式转移承接上取得新突破。组织承办好快餐食品博览会等特色招商活动，加快外向型经济发展，着力引进100个重点招商项目，实际利用境外资金增长5%，实际利用省外资金增长8%，进出口总值增长7%。二是提升项目建设质量。以提高项目开工率、资金到位率、投产达效率为重点，抓住开工、竣工、谋划等关键环节，强化观摩点评、联审联批、督导考核等关键举措，着力推进120个重点建设项目，尤其是总投资195亿元的31个国债资金项目，确保全年新开工项目60个、竣工项目26个，完成投资365亿元。突出战略支撑、引领带动，争取新谋划项目200个以上，增强项目建设接续动力。三是提升企业服务水平。强化问题导向和服务、责任意识，完善市县上下联动和部门横向协调解决机制，支持商会、协会等发展并发挥作用，深入推进银企对接、用工对接、产销对接、产学研对接。积极开展降低实体经济企业成本行动，全面落实国家降低税费负担、财务成本、电力价格、物流成本等减轻企业负担政策措施，为中小微企业发展营造良好环境。选择100家重点企业进行分类帮扶，帮助一批优势企业扩大生产规模和市场份额，稳定“增长点”；帮助一批暂时困难企业恢复正常生产，控制“下拉点”；帮助一批陷入困境企业挖潜增效、盘活资产等途径加快转型突破，减少“出血点”；帮助一批丧失自我修复能力企业通过关停并转、兼并重组、破产清算等方式重获新生，化解“拖累点”，引导支持企业通过内部管理挖潜，实现降成本、增效益。

　　（二）加快结构性改革，提高质量效益。把推进结构性改革特别是供给侧结构性改革作为战略重点，深化全国质量强市示范市创建成果，在适度扩大总需求的同时，有效化解过剩产能，持续减少房地产库存，合理配置要素资源，降低企业成本，扩大公共产品和服务供给，着力去产能、去库存、去杠杆、降成本、补短板；同时，进一步强化科技创新，推进产业优化重组，大力发展实体经济，统筹经济社会协调发展，持续保障改善民生，为发展增活力、增动力、增效益、增均衡、增信心。一方面创新发展现代工业，加快培育链条健全、规模合理、协作高效的产业集群，提高产品附加值和产业竞争力。清洁能源与新材料产业，力争年产350万吨重油煤焦油和工业气化岛项目开工，扎实推进华塑高分子新材料产业园等20个重点项目、完成投资79亿元以上，力争主营业务收入突破1000亿元。绿色食品产业，筹备用好食品产业发展投资基金，研究设立食品产业集团、快餐食品研究院，建成投用市食品安全检（监）测项目，扎实推进年产50万吨中式营养快餐等7个重点项目、完成投资12亿元以上，力争主营业务收入达到600亿元。汽车零部件与电子电器产业，积极发挥鹤壁汽车电子产业集团引领作用，加快汽车零部件与金属镁、军民产业融合发展，扎实推进年产400万套国际高端出口电线束等13个重点项目、完成投资37亿元以上，力争主营业务收入达到200亿元。镁精深加工产业，培育壮大河南镁业公司、河南镁产业联盟及金属镁企业联合体，加快组建市镁应用技术研究院，推进军工超高强镁合金等项目落地和军民融合产业园建设，扎实推进年产2000吨镁合金薄板等3个重点项目、完成投资4亿元以上，力争主营业务收入达到80亿元。现代家居产业，打造智能家居、特色陶瓷、家居型材等产业联盟，完善研发设计、仓储物流、网络服务等配套功能，壮大现代家居、新型陶瓷、智能灯具等专业园区，扎实推进年产84万套星级家具及整体橱柜等8个重点项目、完成投资28亿元以上，力争主营业务收入达到140亿元。同时支持纺织服装服饰等产业提档升级，提高竞争能力。另一方面提速发展现代服务业，坚持传统服务业提升和现代服务业突破并重，持续扩大规模、提升水平，第三产业增加值增长9%左右。新型物流业，突出冷链、煤炭、电商、保税等特色物流，积极搭建发展、供需、融资、人才等服务平台，发挥好服务业领军企业示范引领作用，提升信息化、智能化、差异化发展水平，煤炭产业园区等6个重点项目完成投资20亿元以上，谋划建设快递电商产业园。休闲旅游业，持续推进旅游资源整合、产品策划、提质增效等工作，加快淇河生态区、浚县古城、旅游综合体、文化旅游风情街等建设，9个重点项目完成投资47亿元以上，组织举办好精品节会，接待游客、旅游总收入分别增长15%和20%。网络经济，深入实施“互联网+”行动方案，加快推进电子商务进农村、进社区、进企业，引进建设一批有特色、有品牌的线上线下结合实体店，打造融体验、营销、售后服务为一体的完整产业链，抓好淇县电子商务进农村示范县、邮政电商孵化园等建设，6个重点项目完成投资19亿元以上，积极申创电子商务示范市。文化创意产业，积极发展网络文化、数字服务等新兴产业，促进文化创意与文艺演出、文化娱乐和特色民间工艺等融合发展；依托山城区石林、浚县古城等资源，推进文化旅游、影视拍摄一体化发展。健康养老产业，推行医养结合，加快健康养老产业示范园区和居家养老服务信息平台等建设，谋划建设健康服务与休闲旅游、体育健身等融合发展的健康养生基地，总投资20亿元的养老园区、医疗养老中心完成投资7亿元以上，增强养老服务能力。同时积极发展信息服务、现代金融、商务会展、现代商贸等服务业。

　　（三）提升农业水平，促进稳产增效。加快整建制国家现代农业示范区和国家农业科技园区建设，促进一二三产业深度融合发展，保持农业现代化综合发展水平在全省乃至全国领先地位。一是加快转变农业发展方式。大力发展特色农业、高效农业、绿色农业和品牌农业，在鹤浚快速通道周边、淇河沿岸、南水北调工程两侧，建设一批都市生态观光农业园区；在西部山区、丘陵地带大力发展特色种植养殖业，培育古朴村落、美丽田园、农耕文化等特色旅游和休闲观光品牌。加强农业生态环境保护与治理，探索建立“一控两减三基本”现代生态循环农业发展体系和农业可持续发展长效机制。积极创建三产融合发展示范县。二是提高农业物资装备和技术水平。扎实推进整建制粮食高产创建、高标准粮田建设提升等，完成81个万亩示范片高产创建任务，新建高标准粮田16万亩，深入开展绿色增产模式攻关，提高粮食综合生产能力。积极创建现代农业气象示范市，争取国家农业气象人工气候室项目落地，抓好节水灌溉等农田水利基本建设，增强农业防灾减灾能力。深入推进农机农艺融合，建设特色农业机械化示范基地，提升耕种收综合机械化作业率。三是完善农业经营体系。稳定农村土地承包关系，引导土地合理有序流转，土地适度规模经营达到45%。强化新型职业农民技能、实用技术培训，培育支持农民专业合作社、家庭农场等新型农业经营主体，增强合作社装备、作业服务等能力，新发展农民专业合作社50家、家庭农场100家。健全农业社会化服务体系，提高农业综合标准化服务水平，积极创建全国农业社会化服务综合标准化示范市。四是壮大现代农业产业化集群。发展壮大优质粮食、优质畜禽、良种育繁推一体化、特色农产品加工等产业，加大无公害农产品、绿色食品创建力度，新发展省级产业化集群和示范集群各1个、无公害粮田2万亩、小麦种子繁育基地1万亩、市级以上龙头企业7家，建设一批品牌优势突出、高质高效的现代农业产业化集群，壮大农业产业化龙头企业，组建现代农业产业联盟。五是强化信息科技支撑。加快中国智慧农业研究院、农业硅谷产业园等建设，推动互联网与农业加速融合，大力发展“互联网+农业”。提高农业物联网应用水平，新增益农服务社100家，在一批蔬菜标准园建成农产品质量安全监管和智能物联系统。整体推进农业农村信息化示范基地建设，11个基层农技推广区域站全面建成投用信息服务平台。完善农产品流通设施和市场，强化农资市场监管。

　　（四）加快城乡建设，统筹一体发展。坚持以人的城镇化为核心，突出全域统筹、协同联动，健全城乡一体化发展体制机制，争创国家新型城镇化综合试点，不断改善城乡人居环境、提升城市竞争力。一是加快新型城镇化建设。推动淇滨区城区与开发区、淇水湾片区联动建设都市功能区，建设好高铁广场城市综合体、电子新城核心区、高速高铁以东片区，促进中心城区组团式发展。加快鹤淇一体化进程，努力打造功能统一协调、有机相连、特色鲜明的城市核心区。统筹提升老城区建设发展，支持山城区申报开展全国城区老工业区搬迁改造试点、建设资源型城市转型发展先行区，支持鹤山区深化综合改革实验区建设发展，继续完善老城区基础设施和公共服务设施。推进浚县老城改造提升和新城规划发展，增强综合承载能力，壮大县域经济。完善重点镇配套设施，加快建成一批现代化小城镇。稳妥实施城中村、城郊村城市化改造，按照城市标准建设管理新型社区。二是加快美丽宜居乡村建设。坚持产业、村庄、土地、公共服务和生态规划“五规合一”，因地制宜推进新农村建设和乡村经济发展，增强产业支撑能力和发展活力。高标准规划建设美丽乡村示范带、城乡一体化示范镇、美丽乡村和农村人居环境示范村，力争60%以上村庄建成农村人居环境达标村、20%以上村庄建成示范村。科学保护利用传统村落、民居和历史文化名村名镇，进一步激发生机与活力。三是加快农业转移人口市民化。落实完善农业转移人口市民化政策体系，推动具备条件有意愿的农业转移人口举家落户城镇，努力缩小户籍人口城镇化率与常住人口城镇化率的差距。落实居住证制度，加快村改居步伐，努力实现城镇常住人口全部享有基本公共服务。探索建立农业转移人口市民化成本分担机制，维护好进城落户农民土地承包权、宅基地使用权、集体收益分配权等。四是加快完善城乡基础设施。确保大海线、鹤浚快速通道、东大线改扩建工程竣工通车，开工新老区快速通道拓宽改建工程，力争新老区市域铁路开工，建设农村公路230公里，持续优化调整公交线路和公共停车场，新增一批新能源公交车和公共自行车，加大公共自行车向沿淇河风貌带和老城区投放力度，方便市民出行。将海绵城市建设理念融入城市建设全过程，争取开工海绵城市项目206个、完成投资23亿元，探索建立标准规范体系，高标准完成海绵城市试点市年度建设任务。统筹推进地下综合管廊规划建设，启动钜桥南污水处理厂项目，建成投用鹤淇电厂配套供热管网，市区新增供热覆盖面积200万平方米。五是加强城乡规划管理。做好专项规划编制、城市详细规划和城市设计完善等工作，完成县、乡镇总体规划编制。提高城市和县城精细化规范化数字化管理水平，规范提升城市管理执法和社区物业服务，整治提升公共厕所、农贸市场、道路及配套设施、消防安全等，持续治理脏乱差，加快创建国家卫生城市。积极推进智慧城市创建工作，充分发挥城市智慧平台作用，更好服务群众生产生活。强化重点区域和路段交通整治，完善电子警察、交通信号灯等设施，做到安全有序文明出行。

　　（五）夯实载体平台，提高支撑能力。完善服务功能，创新体制机制，提升发展水平，切实增强综合承载能力。一是推进产业集聚区提质转型创新发展。树立集群、创新、智慧、绿色理念，加快由规模扩张向量、质并重转变，由要素高强度投入驱动为主向投资、创新双驱动转变，着力提高土地投资强度和产出效益。培育壮大优势产业集群，重点打造清洁能源与新材料、绿色食品2个千亿级，汽车零部件与电子电器500亿级，镁精深加工、现代家居2个超百亿级产业集群。积极引进设立产品、产权交易平台等，着力解决产业配套、金融支持、土地保障、人力资源等突出问题，全面提升集聚产业、企业、人才、资金等服务能力水平。落实完善产业集聚区与行政区域管理套合体制机制，推动产业集聚区成规模、提层次、上星晋位，力争省级产业集聚区完成固定资产投资449亿元、实现主营业务收入1300亿元、完成规模以上工业增加值285亿元，争取新增1个二星级产业集聚区。二是推进服务业“两区”扩容增效。坚持促集聚、育特色、提服务、强融合，商务中心区重点吸引企业总部、研发中心、营销中心、电商服务平台等功能性机构入驻，加快二次招商步伐，提高签约入驻率，力争进入省“十强十快十先”行列；特色商业区以龙头引进带动整体开发，持续做大特色产业规模。培育壮大服务业集群，制定年度“两区”建设专项方案，加快国家863科技创业园等亿元以上项目建设，确保“两区”完成投资50亿元以上。三是推进口岸功能完善提升。完善海关、出入境检验检疫局功能，加强与“一带一路”沿线城市和沿海港口城市关检合作，健全大通关服务体系，提高通关便利化水平。做好综合保税区申建和国际商品城相关工作，积极打造全省一流、辐射豫北的区域性开放平台。

　　（六）深化改革创新，增强内生动力。坚持把改革创新作为增动力、添活力的核心抓手，更加精准地对接发展所需、基层所盼、民心所向，确保一批具有标志性、引领性、支撑性的改革创新任务取得实质性突破。改革方面：一是深化市场配置要素改革。全面清理限制非公有制经济发展的不合理规定，鼓励、支持、引导非公有制经济发展，更好地保护各种所有制经济产权和合法权益。在公共服务产品供给方面，政府能通过购买服务获得的不再直接承办，能与社会资本合作的吸引社会资本参与。进一步深化商事制度改革，放宽市场准入。二是深化投融资体制改革。探索建立企业投资项目负面清单制度，创新政府和社会资本合作项目收益分配机制，进一步释放民间投资潜力和活力。对市属国有企业实施功能分类和职能定位，健全管理体制机制，完善法人治理结构，组建鹤壁国有资本投资运营集团；强化国有资产监管职责，建立国有资产出资人监管权力和责任清单，以管资本为重点加强国有企业监管，防止国有资产流失。建立健全以政府债券为主体的政府举债融资机制，防范债务风险。整合优化全市投融资平台，壮大市级投融资平台规模，提高市、县区协同投融资能力。三是深化财税体制改革。健全政府预算体系，加大预算统筹力度，推进预算公开透明，建立跨年度预算平衡机制，完善市本级财政专项资金管理办法。落实营改增试点和消费税、资源税费改革工作，做好税收征管体制综合改革工作。四是深化农村改革。基本完成农村土地承包经营权确权登记颁证、农村宅基地和集体建设用地使用权确权登记发证工作。探索不同层级整合涉农资金的有效方式，积极开展农民以土地经营权入股农民合作社和农业产业化龙头企业试点，引导农村土地经营权有序流转，加快建设农村土地流转试点。创新方面：扎实推进国家可持续发展试验区、全国创新驱动助力工程示范市建设，启动“互联网+”研究院、大数据创新空间等二期项目，力争新建科技创新园、众创空间、科技企业孵化器等4家，增强协同创新能力。新建产业技术研究院和产业技术创新战略联盟4家，实施8项重大科技专项，突破5项关键核心技术，增强产业创新能力。新建工程技术研究中心10家、院士工作站1家，培育高新技术企业和“科技小巨人”13家，引进培养一批科技创新创业人才和团队，增强企业创新能力。加快推进与中科院宁波材料所、化学研究所等合作共建创新研发平台建设，争取在谈的4所科研院校合作尽快取得实效，持续扩大科技开放合作。探索建立科技创业投资基金，组织开展好科技保险、知识产权质押贷款试点推广工作，深化大众创业万众创新，强化创新突破的好趋势。要素保障方面：深化与域外银行、保险机构、投资集团的合作，加大引进市外金融机构力度；鼓励支持企业扩大直接融资规模，发挥好中小企业还贷周转金作用，做好农信社改制组建农商行工作；实施信贷业务主办银行、金融机构抱团帮扶等制度，提高信贷审批效率。强化农民工职业技能提升和企业家梯次培养，完善人才引进配套政策，加快高层次管理人才、技术工人和科研型人才引进培育，缓解劳动密集型企业招工难现象。建设好国家首个国土空间优化发展实验区，做好永久基本农田划定和土地利用总体规划调整，推进不动产统一登记工作，依法规范和节约集约使用土地，推进城镇低效用地再开发和工矿废地复垦，控制农村集体建设用地规模。

　　（七）提升生态成效，建设美好家园。深入实施蓝天、碧水、乡村清洁工程，确保空气优良天数、淇河水质等主要环境指标保持全省前列，让鹤壁的天更蓝、地更绿、水更清、人与自然更和谐。推动绿色发展成品牌。巩固提升国家森林城市创建成果，开展“五林共育”行动，建好防护林、培植用材林、发展经济林、扩展能源林、保护特用林，完成造林和补植补造18万亩。顺应自然保育生态，整治提升淇河、汤河、卫河等河流，加快国家湿地公园、淇河下游湿地公园等建设，使淇河永葆生机活力；开展“五水共治”行动，治污水、防洪水、用雨水、保供水、抓排水，完成棉丰渠、天赉渠、二支渠、护城河等水系改造工程量的60%，努力消除黑臭河。坚持数量和质量并重、质量优先，开展“五边共美”行动，提升公路边、铁道边、河流边、山坡边、街巷边的绿化、洁化和美化水平，加快创建国家园林城市，使适宜绿化的地方都绿起来。推动低碳发展上水平。大力推进国家节能减排财政政策综合示范市建设，力争示范项目完成投资15亿元以上，推动落后产能淘汰和传统产业节能减排改造。开展“五气共管”行动，着力降工业废气、控燃煤烟气、减汽车尾气、消城市扬尘、除油烟废气，高标准完成重点行业脱硫、脱硝、除尘和清洁生产改造，有效改善大气环境质量。持续开展农村人居环境综合整治，加强种植、畜禽养殖等农业面源污染防治，推进土壤污染治理及修复，农作物秸秆全量化综合利用、秸秆综合利用率保持在95%以上。推动循环发展提层次。围绕创建全国循环经济示范市，出台实施《鹤壁市循环经济条例》，完善循环经济农业、工业、服务业和社会体系，注重技术研发和标准制定，加快实现由提供产品向提供技术、标准、服务转变。推进宝山产业集聚区废物综合利用、能量梯级利用等项目建设，推广企业循环式生产、产业循环式组合、园区循环式改造，提高资源、能源产出率。积极发展节能环保产业和再生资源综合利用产业，加快建设垃圾分类、收运体系等废旧物资资源化利用示范项目，构建覆盖全社会的资源循环利用体系。健全生态保护制度。完善生态环境损害责任追究及生态文明绩效考核制度，落实排污单位主体责任和行业管理部门监管责任，实行一票否决并严肃追究怠政失职行为。加强污染物排放总量控制，探索建立排污权、碳排放权初始分配制度，培育和发展交易市场。在环境高风险领域探索建立污染强制责任保险制度，引入第三方治理。完善生态环境突发性灾害事件应急机制，探索设立节能减排投资引导资金。开展环保综合督查和重点领域环境专项执法检查，严厉打击各类环境违法行为，清理整顿环保违法违规建设项目。做好环保机构监测监察执法垂直管理体制改革。开展“五节共推”行动，做好节能、节水、节地、节材、节矿和能效、水效领跑者引领工作，倡导绿色低碳循环发展方式和生活方式，建设节约型社会。

　　（八）注重传承创新，打造文化强市。以社会主义核心价值观为引领，以创建省级文明城市为抓手，全面提升文化供给能力和服务质量，推动物质文明、精神文明协调发展，积极创建国家公共文化服务体系示范区，提升文化软实力。一是加强精神文明建设。深入开展中国梦和社会主义核心价值体系宣传教育，传承中华传统美德，大力弘扬“鹤壁好人”精神，在全社会形成向善向好良好风尚。深化群众性精神文明创建活动，构建学校、家庭、社会三位一体的未成年人思想道德教育网络，倡导全民阅读，提高全社会诚信意识和文明程度。加强互联网舆论引导与管理，净化网络环境。二是加快发展文化事业。市示范性综合实践基地和市综合活动中心建成投用，市图书馆新馆、市档案馆实现搬迁，开工建设市科技馆、城市规划展览馆、鹤壁好人展示馆，统筹推进乡镇综合性文化服务中心、村级综合活动场所等建设。全民健身活动中心暨体育公园体育场馆主体完工，市体育中心启动建设。实施好常态化文艺演出、公共文化进基层、文化设施免费开放等惠民工程。加强文物保护利用和非物质文化遗产保护传承，做好大赉店遗址环境整治和大运河、黎阳仓遗址保护展示等工作。三是培育壮大文化产业。推进文化与科技、金融、旅游、互联网等融合发展，传统媒体和新兴媒体融合互动。围绕民俗文化、淇河文化、殷商文化、军事文化、诗歌文化等，大力引进知名文化企业，促进新型文化业态发展。提升民俗文化节、文博会、环淇河自行车赛等层次水平，打造一批特色活动和艺术精品。办好市十届运动会，广泛开展全民健身活动，提高竞技体育水平，加强心理健康服务。结合“中原文化丝路行”，扩大对外文化交流和贸易。四是深化文化体制改革。加大政府购买公共文化服务力度，积极吸引社会资本投入，探索建立公共文化服务城乡联动机制，加快经营性文化事业单位转企改制步伐，深化公益性文化事业单位体制机制改革，创新文化传播方式方法。

　　（九）创新社会治理，保障改善民生。从落实群众最关心最直接最现实的重点民生工程入手，全面提高公共服务共建能力和共享水平，切实保障人民生命财产安全、保障人民生活改善、保障人民身体健康，增强获得感和满意度。打好脱贫攻坚战。落实扶贫政策，创新开发方式，夯实脱贫基础，补齐贫困短板，启动20个贫困村整村推进扶贫开发、2000名西部山区贫困群众扶贫搬迁，力争1.5万贫困人口稳定脱贫；完成盘石头水库第三期移民搬迁，做好移民后期扶持工作。扩大就业增加收入。坚持以创业创新带动实现更高水平的就业，完成职业技能培训3万人次以上，新增农村劳动力转移就业1.5万人，做好重点群体、困难人员就业工作，妥善解决化解过剩产能中下岗分流职工再就业问题，动态消除零就业家庭。健全企业工资集体协商、正常增长和支付保障机制，构建和谐劳动关系；适当提高机关事业单位人员基本工资，落实县以下机关公务员职务与职级并行制度和乡镇工作补贴；多渠道增加城镇中低收入者和农民收入。提高社会保障水平。全面实施全民参保登记计划，扎实做好城镇职工基础养老金全国统筹和基本医疗保障政策全省统一相关工作。落实完善养老保险、医疗保险、社会救助、社会福利等制度。深化机关事业单位养老保险制度改革，继续提高企业退休人员基本养老金。新农合和城镇居民医疗保险人均补助标准提高到410元，职工医保、城镇居民医保政策范围内住院费用支付比例分别提高到80%以上、75%左右，适当提高大病保险人均筹资水平和支付比例，探索建立城乡大病保险向贫困对象、高额医疗费用患者倾斜机制，积极推进省内异地就医即时结算。增强城乡低保、医疗救助、临时救助保障能力，城乡低保财政补差水平月人均分别提高15元和12元，农村五保对象集中、分散供养标准分别提高到年人均4000元和3000元，全面实施临时救助制度。全部建成县级社会福利中心，实施残疾人帮扶活动，对6岁以下贫困残疾儿童实施抢救性康复，全面建立困难残疾人生活补贴和重度残疾人护理补贴制度，建立经济困难高龄、失能老人补贴制度，健全农村留守儿童、妇女、老人关爱服务体系。发展红十字事业，鼓励慈善捐赠和志愿服务，让困难群众感受到更多温暖。增强住房保障能力。以去库存、促消费为重点促进房地产业健康发展。将有稳定劳动关系的常住人口纳入公积金覆盖范围，促进新市民在城镇购房，鼓励农民进城购房。建立购租并举住房制度，提高棚改货币化安置率，培育发展住房租赁市场，主要通过货币化补贴、政府购买或长期租赁商品房解决公租房新增需求。基本建成各类保障性住房5000套、新开工595套，改造农村危房1500户。统筹教育均衡发展。以办好人民满意的教育为目标，实行城乡统一的义务教育学校公用经费基准定额，年生均经费小学达到600元、初中达到800元，对寄宿制学校按照年生均200元标准增加公用经费补助。以解决“入园难”“大班额”等为重点，新建改扩建幼儿园19所、建设中小学5所、改造薄弱学校12所，全市中小学超大班减少20%、大班减少10%。深入实施职业教育二期攻坚工程，推进省级职业教育品牌示范校和特色校创建、国际化职业教育合作，提升办学层次和本科办学水平。以完善助学资助体系为保障，免除建档立卡家庭经济困难普通高中学生学杂费，让贫困家庭孩子有机会接受更好教育。启动新一轮农村义务教育学校布局调整，加强农村寄宿制学校建设，实施乡村教师支持计划。支持和规范民办教育发展，办好特殊教育。改进公共卫生服务。深化医药卫生体制改革，提升人民医院、中医院等对外开放合作水平，抓好三甲医院创建、县级公立医院综合改革和乡镇卫生院、社区卫生服务中心、标准化村卫生室等建设，扎实有序推进分级诊疗。推进基本公共卫生服务均等化，人均基本公共卫生服务经费补助标准提高到45元，加强重大传染病、慢性病、地方病、职业病防治，免费为城乡常住居民提供14种国家基本公共卫生服务。积极引进使用好医疗卫生人才，提升从业人员能力素质，提高医疗卫生服务水平。坚持计划生育基本国策，落实全面两孩政策，提升出生人口素质。维护社会安全稳定。加强立体化社会治安防控体系建设，依法打击违法犯罪和暴恐活动。强化流动人口服务管理和特殊人群教育、管理、服务、帮扶等工作。

　　注重依法化解社会矛盾，着力解决土地征用、房屋拆迁、农民工工资发放等问题。加强多种形式宣传教育，有效防范化解重点地区、重点行业非法集资问题。严格落实安全生产责任制，深化重点行业、重点领域专项整治，坚决遏制安全生产和食品药品安全等重特大事故发生。加强基层基础建设，深入推进城乡社区网格化管理，促进社会治理多元化参与，构建全民共建共享的社会治理格局。

　　加强国防教育和国防后备力量建设，支持驻鹤解放军、武警部队、民兵预备役和人民防空建设，进一步解决好部队和民兵预备役建设遇到的困难问题，深入开展双拥共建，巩固和发展军政军民团结，促进军民深度融合发展。支持工会、共青团、妇联等人民团体发挥桥梁纽带作用。继续做好民族宗教和外事侨务、对台、统计、档案、史志、地震、人防等工作。

　　四、建设高效 廉洁政府

　　坚持为民务实清廉，统筹推进法治政府、创新政府、廉洁政府和服务型政府建设，不断提升治理能力和服务水平，打造最优发展环境和从政环境。

　　加快转变政府职能。深入推进简政放权、放管结合、优化服务工作，着力从重数量向提高含金量转变。深化行政审批标准化工作，再精简一批行政审批事项、涉审批中介服务事项，落实好权责清单制度，全面梳理、依法公开、动态调整部门及相关中介、国有企事业单位公共服务目录清单。完善推广随机抽查、规范事中事后监管制度，切实把该管的管好。改进政府服务方式，探索推进市县区行政审批一体化改革和“互联网+政务服务”，完善行政服务和公共资源交易中心功能，方便企业和群众办事。

　　深入推进依法行政。健全重大行政决策机制，促进依法科学民主决策。认真落实重大事项向市委请示、向人大报告和向政协通报制度，拓宽参政议政渠道，广泛听取民主党派、工商联、无党派人士、人民团体意见，切实办理好人大代表建议和政协提案。自觉接受人大法律监督、工作监督和政协民主监督，高度重视司法监督、舆论监督和社会监督，不断完善政府内部层级监督。深入推进服务型行政执法，全面落实行政执法责任制，扎实推进综合行政执法体制改革试点，促进严格规范公正文明执法。启动“七五”普法。深化政务公开，让行政权力始终在法治轨道和阳光下运行。

　　持续改进工作作风。认真贯彻落实八项规定精神，自觉践行“三严三实”要求，扎实开展“双学一做”，全面加强政府系统作风建设，驰而不息纠正“四风”。健全岗位履职责任制和激励机制，引导支持各级公务人员敢作为、能作为、善作为，持续开展懒政怠政为官不为专项治理，提高执行力和行政效能。强化教育培训和实践锻炼，深入开展调查研究，着力提高贯彻新发展理念的能力水平，提升公务人员的积极性主动性创造性。完善督促检查、纠错问责机制，打通政策落实的“中梗阻”及“最先最后一公里”。

　　切实加强廉政建设。严明政治纪律，严守政治规矩，严格正风肃纪，使广大干部做到忠诚干净担当。全面落实党风廉政建设责任制，加强对政府系统工作人员的党规政纪教育。强化行政监察、审计等工作，以零容忍态度惩治腐败，坚决查处权力寻租、权钱交易和损害群众利益等行为，着力解决群众身边的不正之风和腐败问题。坚持厉行节约、反对铺张浪费，严格规范财政资金管理使用，以廉洁从政和反腐倡廉实效保障发展、取信于民。

　　各位代表，实干托起希望，奋斗铸就辉煌。让我们在省委、省政府和市委的正确领导下，以奋发有为的精神状态、攻坚克难的拼搏意志、只争朝夕的紧迫劲头，开拓创新，担当作为，实干争先，为在全省率先全面建成小康社会、奋力加快更高水平生态活力幸福之城建设进程，为中原更加出彩而努力奋斗！
